# Prediction of protein p*K*_a_ with representation learning†

**DOI:** 10.1039/d1sc05610g

**Published:** 2022-02-01

**Authors:** Hatice Gokcan, Olexandr Isayev

**Affiliations:** Department of Chemistry, Mellon College of Science, Carnegie Mellon University Pittsburgh PA USA olexandr@olexandrisayev.com

## Abstract

The behavior of proteins is closely related to the protonation states of the residues. Therefore, prediction and measurement of p*K*_a_ are essential to understand the basic functions of proteins. In this work, we develop a new empirical scheme for protein p*K*_a_ prediction that is based on deep representation learning. It combines machine learning with atomic environment vector (AEV) and learned quantum mechanical representation from ANI-2x neural network potential (J. Chem. Theory Comput. 2020, 16, 4192). The scheme requires only the coordinate information of a protein as the input and separately estimates the p*K*_a_ for all five titratable amino acid types. The accuracy of the approach was analyzed with both cross-validation and an external test set of proteins. Obtained results were compared with the widely used empirical approach PROPKA. The new empirical model provides accuracy with MAEs below 0.5 for all amino acid types. It surpasses the accuracy of PROPKA and performs significantly better than the null model. Our model is also sensitive to the local conformational changes and molecular interactions.

## Abstract 2

We developed new empirical ML model for protein p*K*_a_ prediction with MAEs below 0.5 for all amino acid types.

## Graphical abstract

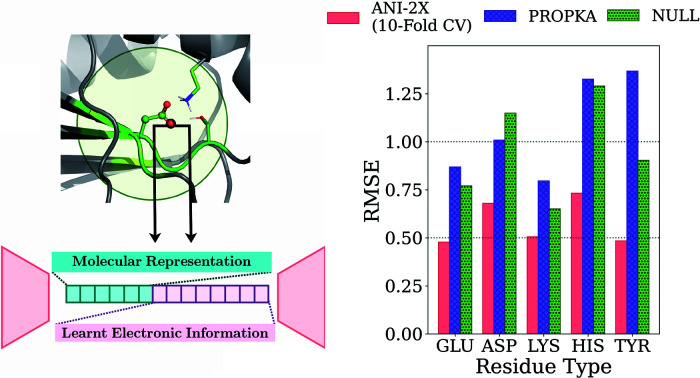

## Introduction

Basic features and the behavior of proteins, such as folding or ligand binding, heavily depend on the environmental conditions like the local protein environment. Titratable amino acids like aspartic acid (Asp) or histidine (His) are essential in many biological processes^1–5^ and can be either protonated or deprotonated depending on the local environment. Thus, determination of the ionization states *via* p*K*_a_ predictions is a prerequisite to understand the protein function. Determination of p*K*_a_ values *via* experimental procedures is challenging and the most reliable results for proteins can be obtained only with NMR titrations.^6^ This predicament enforces the p*K*_a_ predictions in proteins by means of theoretical applications.^7^ There is a tremendous amount of work on theoretical p*K*_a_ calculations in the literature. These approaches can be classified into three categories as (i) microscopic methods,^8,9^ (ii) macroscopic methods which establish continuum electrostatics,^10^ and (iii) knowledge-based methods that rely on empirical parameters.^11,12^

Among the three classes of theoretical p*K*_a_ calculations, microscopic methods such as quantum mechanical (QM) or quantum mechanics/molecular mechanics (QM/MM) approaches are considered the most reliable ones to compute p*K*_a_ values of small molecules.^8,13^ The most traditional approach with QM methods is to employ thermodynamic cycles by computing protonation/deprotonation free energies in the gas-phase and in solution.^14–23^ However, these calculations do not always provide reliable p*K*_a_ values due to reasons such as the instability of the species in the gas-phase or large conformational differences between the gas-phase and in solution.^17,24^ In the case of the proteins, QM approaches are impractical simply due to the system size and can only be achieved with model systems consisting of the local protein environment of the residue of interest. Nevertheless, the size of the model and the choice of the local environment can alter the theoretical p*K*_a_ values.^25^ A more practical microscopic method to compute p*K*_a_ values is the hybrid quantum mechanics/molecular mechanics (QM/MM) approach, in which the titratable residue is modeled at a quantum level. At the same time, the remaining media is treated with molecular mechanics.^26–28^ Molecular dynamics (MD) based methods such as free energy perturbation^29,30^ and constant pH molecular dynamics (CPHMD) simulations^31–41^ can provide reliable p*K*_a_ values for protein residues. Combining enhanced sampling techniques with CPHMD simulations can also improve the accuracy of p*K*_a_ predictions.^34,42–47^ Nevertheless, the need for fast and reliable approaches to predict p*K*_a_ values of protein residues can render the microscopic methods impractical due to the exhaustive computation time.

Macroscopic methods rely on either the numerical Poisson–Boltzmann equation (PBE)^10,48–51^ or the Generalized Born (GB) technique with analytical approximations to electrostatic energies.^52,53^ These methods model the proteins as a homogeneous medium with a low dielectric constant while the environment (solvent) is modeled with a high dielectric constant. The PBE based methods and their variations^54–60^ can allow modeling the accessibility of the solvent to the titratable residues^61,62^ and multiple ionizable residues within the proximity.^63,64^ Even though there are different suggestions for the dielectric constant of proteins that varies from 4 to 80,^65–73^ the appropriate value depends on the polarity of the surrounding residues and the flexibility of the protein.^74,75^ This issue can be addressed by taking the flexibility of the protein into account *via* techniques that involve ensembles of conformers.^54,76–82^ An example of such an approach is the Multi-Conformation Continuum Electrostatic (MCCE) method which has been shown to successfully predict p*K*a values of several protein residues with different force fields.^70,83–85^

Empirical methods are based on statistical fitting of environmental descriptors and parameters to the three-dimensional structures of proteins. Their sufficiently accurate predictions for most cases combined with their low computational cost make them widespread and favorable. There are a variety of empirical tools with comparable accuracies,^86–88^ but PROPKA^11,12^ is the most widely used for protein p*K*_a_ predictions. Conceptually, PROPKA computes the change of the amino acid p*K*_a_ value from water to a protein environment. In this tool, the environmental perturbation is expressed as the sum of perturbation contributions from a protein environment.

Recent studies with machine learning (ML) algorithms for p*K*_a_ estimations of transition metal complexes have provided new empirical schemes.^89,90^ These approaches combine the pattern recognition capabilities of ML algorithms with the atomistic and molecular features that are obtained with a QM tool. However, this scheme can only be practical for proteins if molecular descriptors are obtained with low computational cost, such as neural network potentials (NNPs). Over the last decade, NNPs have been shown to provide accuracy approaching that of QM calculations and comparable computational cost with all-atom force fields. These potentials, such as ANI^91–98^ and AIMNet,^99^ can learn the electronic environment of an atom in conjunction with the many-body symmetry functions that arise from the coordinates.^100,101^ Using this learned information and combining it with the structural fingerprints that depend on the coordinates, NNPs can predict target molecular properties such as energy and forces. Thus, NNPs can be utilized to obtain information that stems from the atomic environment, and this information can be used to train ML models for protein p*K*_a_ estimations.

In this context, we developed an empirical scheme for protein p*K*_a_ predictions that employs ML algorithms for five amino acid types (ASP, GLU, HIS, LYS, and TYR). We rely on representation learning, *i.e.*, learning representation of the data by automatically extracting useful information when the ML model is trained. We used ANI atomistic neural network architecture that learns molecular representation end-to-end, *i.e.*, directly from atomic coordinates. This molecular representation reduces the dimensionality of a molecular structure into a compact vector format that encodes important quantum mechanical information.

## Methods

**Fig. 1:**
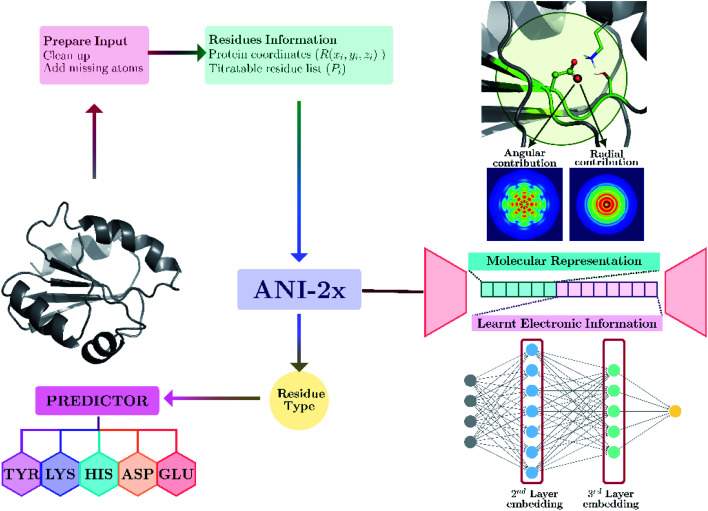
Protein p*K*_a_ prediction with neural network features obtained with ANI-2x. Each amino acid type has its own predictor.

Our model provides predictions *via* the atomic environment and the learned electronic information that is obtained with a widely used NNP, ANI-2x.^96^ The workflow for protein p*K*_a_ prediction is depicted in Fig. 1. In the present work, each amino acid type is treated separately to improve the accuracy by ensuring different molecular features for different amino acid types. Models are trained and tested over hundreds of experimental p*K*_a_ values, and the accuracy is also compared with the widely used PROPKA^12^ tool. The presented approach performs significantly better than null models and improves the current empirical methods for p*K*_a_ estimations.

### Reference data for training

The p*K*_a_ model is trained and tested with two datasets. The first dataset is obtained from the PKAD database.^102^ This dataset consists of over 1500 experimentally measured p*K*_a_ values of residues on both wild type (WT) and mutant proteins. The second dataset consists of 337 entries that were extracted from the primary literature.^103–127^ Mutation of a residue on a protein can cause significant conformational changes that alter the amino acids' electronic environment in proximity to the mutation site. However, not all mutant proteins have crystallographic structures deposited to the databanks. Extensive conformational sampling must be performed to account for the conformational alteration due to the mutations. Since conformational sampling is out of the scope of this study, all mutant protein entries were excluded from datasets. Our model is trained only for WT proteins. This selection results in training and test datasets containing entries from 186 WT PDB structures. The distribution of the p*K*_a_ values in training and test datasets can be found in the ESI (Fig. S.1).† For this initial proof of principle model, only five titratable residues (GLU, ASP, LYS, HIS and TYR) are selected as targets for p*K*_a_ predictions.

### Data curation

Crystallographic structures of 187 WT proteins are obtained from the PDB. A flowchart for data preparation prior to the training can be found in the ESI (Fig. S.2).† In conventional PDB files, the crystallographic structures can involve entries other than proteins and nucleotides, such as ligands, mobile counterions, metal ions, or water molecules. It is important to state that the presence of a co-factor or a ligand can alter the p*K*_a_ of residues within a protein. However, any entry other than proteins and nucleotides is removed from PDB structures due to two reasons. First, the number of atomic species that are defined in a neural network potential (NNP) is currently limited to nonmetals. This limitation prevents inclusion of HETATM entries that can have atomic species that NNP does not define. Second, the conditions in the experimental procedures for p*K*_a_ determination and the crystallographic data preparation can be different. PDB entries correspond to constrained structures obtained using either X-ray or neutron diffractions, requiring specific strategies to achieve crystallographic packing. For example, many PDB entries tend to contain mobile counterions due to the packing procedures and these ions mainly do not exist in experimental p*K*_a_ determinations.

After the clean-up of PDB entries, missing heavy atoms and H atoms are added with the *tleap* module of AmberTools21 ^128^ using the ff14SB force field for proteins^129^ and BSC1 force field for DNA.^130^ For titratable protein residues, standard protonation states are assumed. To prevent any possible steric clashes after the addition of missing atoms, very short gas-phase minimizations (250 steps of steepest descent followed by a conjugate gradient up to 500 steps in total) are performed using the *sander* module of AmberTools21.^128^

### Descriptor calculations

Minimized structures are used as inputs for NNP to compute all descriptors. A detailed description of ANI neural network potential and corresponding descriptors can be found elsewhere.^96,100^ Briefly, in ANI-type NNPs, the environment of the atomic species in the given coordinate system is transformed into atomic environment vectors (AEVs) that contain radial and angular contributions (see Fig. 1). Since the p*K*_a_ of amino acids in proteins are sensitive to the neighborhood environment, naturally, AEVs were chosen as candidates for p*K*_a_ descriptors. This representation includes structural information on both bonded and non-bonded interactions of any given atom within the default ANI cutoff distance (*r*_cut_ = 5.2 Å). In addition to AEVs, neural network embeddings were chosen as learned representations. Therefore, 2^nd^ and 3^rd^ layers of atomic neural network embeddings are selected as additional descriptor candidates.

### Feature importance and training

**Fig. 2:**
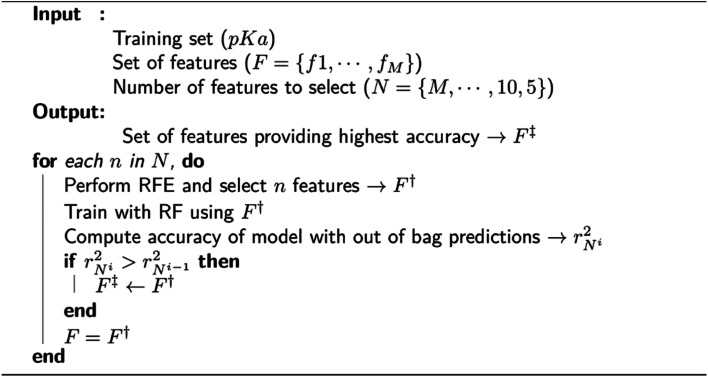
Pseudo-code for feature selection with recursive feature elimination (RFE).

We observed that many features in the overall descriptor were redundant or highly correlated. To eliminate the redundant features, a three-step filtering procedure is adopted. First, noninformative features (values of 0.0) for all reference data are removed. Second, correlation of the features is computed, and highly correlated features (correlation coefficient > 0.95) are eliminated. Third, a recursive feature elimination (RFE)^131^ process is performed using a random forest regressor (RF)^132^ algorithm as implemented in the scikit-learn package.^133^ RFE is a technique that allows defining the least important features using an importance ranking, and it has been shown that ML models benefit from it.^134^ The pseudo-code for RFE is depicted in Fig. 2. In each recursive step of the procedure, the feature importance is measured, and a desired number of features are kept (*F*^†^) by removing less important ones. The new feature list is used to perform training with RF using 1000 decision trees. A final set of features (*F*^‡^) is defined by the local model that has the best coefficient of determination for predictions over out-of-bag samples. After obtaining the final set of features, a 10-fold cross-validation (CV) is performed with RF using same settings for training in the feature elimination process.

### Molecular dynamics simulations and clustering

Two different ionization states of ASP26 (neutral: ASH, and negatively charged: ASP) on human thioredoxin conformer (PDB ID: 3TRX) are considered. Topology and coordinate files are built with the default ionization states for residues in the ff14SB force field for proteins^129^ using the *tleap* module of AmberTools21.^128^ The samples are neutralized using Na^+^ counter ions: 4Na^+^ for the sample containing neutral ASP, and 5 Na^+^ for the sample containing negatively charged ASP. To provide salt concentration, 5 Na^+^ and 5 Cl^−^ counter ions are added to the samples. Waters in the original crystal structure are deleted, and the samples are solvated using TIP3P water molecules^135^ with a distance between the solute and the edge of the box being 12 Å, which results in an average box dimension of 66.8 Å × 69.7 Å × 62.3 Å.

Simulations are performed using the CUDA version of AMBER20's *pmemd* module.^128,136,137^ A time step of 1.0 fs is used along with Berendsen temperature coupling^138^ and SHAKE algorithm^139^ for the bonds involving hydrogen atoms. The particle mesh Ewald summation (PME) technique^140^ is employed using a cutoff distance of 8 Å. We carried out an 11-step equilibration procedure^141^ that consists of harmonic restraints on protein residues and its reduction in each step at 10 K, which is followed by the gradual heating of samples to 300 K with a gradual harmonic restrain reduction at 300 K. A 50 ns long production simulation is performed using equilibrated samples for both samples. Production trajectories are used to cluster the frames using a hierarchical agglomerative (bottom-up) approach as implemented in the *cpptraj* module of AMBERTools21.^128^ Clustering is performed using the root mean square method as the distance metric for the carboxyl group of the ASP26 side chain (ASH26 in the case of neutral ASP). It is finalized when the minimum distance between the clusters is larger than 1.5 Å. The best cluster representatives are selected using the lowest cumulative distance to all the other frames in the same cluster.

## Results and discussion

There has been a surge of approaches looking to learn a representation that directly encodes information about molecules.^142,143^ The idea behind representation learning is to learn a mapping that embeds molecular structures as points in a low-dimensional vector space.^144^ The goal is to optimize this mapping so that relationships in the embedding space reflect the similarities between objects. After optimizing the embedding space, the learned embeddings can be used as feature inputs for downstream machine learning tasks. The key distinction between representation learning and traditional descriptor calculations is how they treat the molecular structure problem. Descriptors treat this problem as a pre-processing step, using domain knowledge and hand-crafted rules to extract molecular information. In contrast, representation learning treats this problem as a machine learning task, using a purely data-driven approach to learn embeddings that encode a molecular structure.

The p*K*_a_ of an amino acid on a protein can be affected by different environmental features such as amino acids in proximity or solvent access. The surrounding amino acids can be encoded through so-called atomic environment vectors (AEVs) which can be obtained with popular atomistic neural network potentials like ANI.^96^ Even though the presence of the solvent cannot be modeled with the current ANI-2x implementation, the gas-phase electronic-structure contributions can be addressed with neural network embeddings. These embeddings would provide information regarding the electronic environment of the titratable residue.

**Fig. 3:**
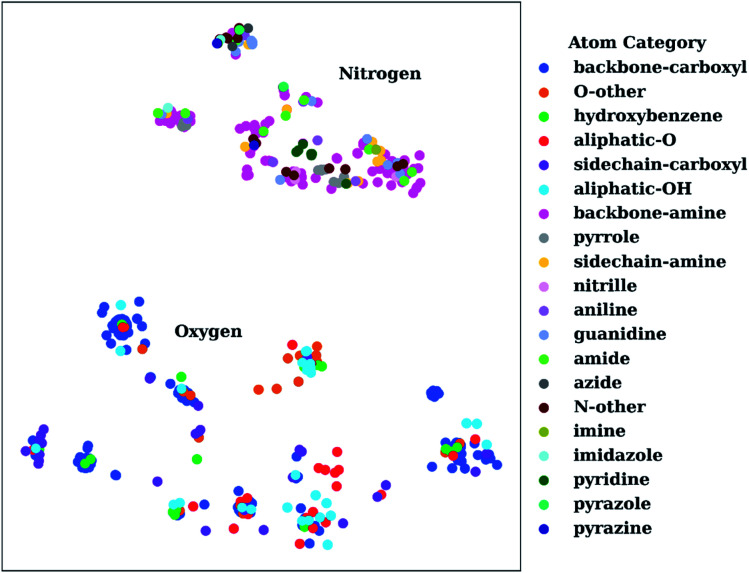
*t*-Distributed stochastic neighbor embedding (*t*-SNE) maps depicting the similarity of 3^rd^ layer neural network embeddings for oxygen and nitrogen atoms located on structures from the SwissSidechain database.^145^ The backbones of the corresponding structures ensure a zwitterion form with NH_3_^+^ (backbone-amine) and COO^−^ (backbone-carboxyl) as backbone groups.

To show the utility of the representation learning, we first performed a simple exercise. We extracted 3D structures for 171 natural and non-natural amino acids from the SwissSidechain database.^145^Fig. 3 shows a 2D *t*-distributed stochastic neighbor embedding (*t*-SNE)^146^ projection of atomic embeddings for oxygen and nitrogen atoms based on the 3^rd^ (top) layer neural network. Naturally, oxygen and nitrogen atoms show two distinctly different clusters corresponding to each element.

Inside the oxygen cluster, titratable groups like sidechain carboxyls, aliphatic and aromatic alcohols are spread out. This is possible due to the very different environments modulated by non-natural amino acids. We hypothesized that the difference in embedding vectors should reflect the acid–base properties of these groups too. Therefore, these embedding vectors could be used as descriptors for empirical p*K*_a_ prediction. For the sake of completeness, we will consider all possible descriptors, *i.e.*, AEV, and 2^nd^ and 3^rd^ layer neural network embeddings obtained with the ANI-2x model as an initial set of descriptors.

To assess the performance of ML models with ANI-2x descriptors, the available p*K*_a_ data are divided into training and test subsets. Different ML algorithms were tested, and the accuracies were analyzed. Results obtained with different procedures are depicted in the ESI (see Fig. S.3).† We observed that linear regression (LR) and support vector machines (SVMs) with linear kernel yielded similar results. Training with the RF provided more accurate results with MAEs of about 0.5, while the inclusion of recursive feature elimination (RFE) improved the accuracy even further. RFE resulted in a feature space of about 10 to 100 descriptors for amino acids. We observed that the features belong to the side chains and the features that belong to the backbone atoms are selected as important descriptors. This can be related to the learned inductive (through-bond) effects. Feature elimination revealed that even though most of the descriptors from the initial feature list are eliminated, all the feature classes are preserved in the final feature list. These results indicate that p*K*_a_ predictions require the information regarding the atomic environment of titratable residues and electronic information encoded by the neural network embeddings of the NNP.

**Fig. 4:**
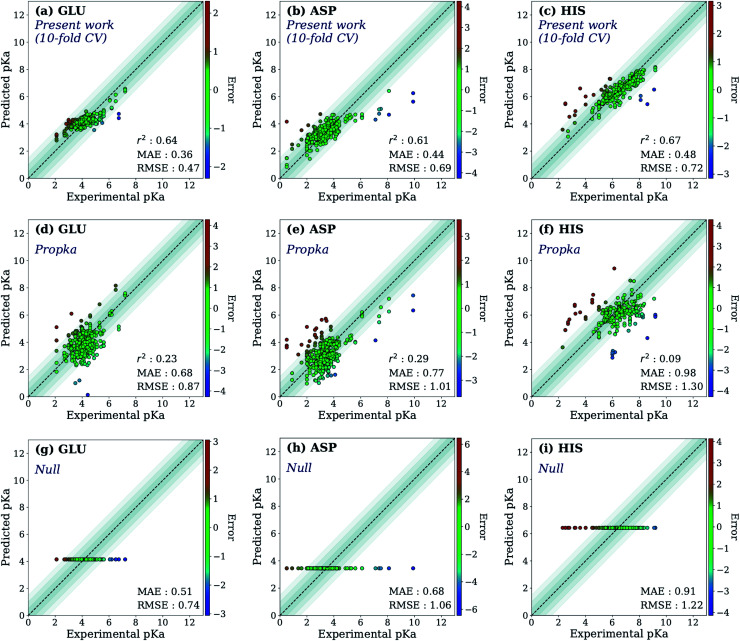
The accuracy of the predictions of experimental p*K*_a_ values for (a) 10-fold cross-validation predictions with the ML model for GLU, (b) 10-fold cross-validation predictions with the ML model for ASP, (c) 10-fold cross-validation predictions with the ML model for HIS, (d) GLU using PROPKA, (e) ASP using PROPKA, (f) HIS using PROPKA, (g) GLU with null model, (h) ASP with null model, (i) HIS with null model.

First, the model accuracy was accessed with *k*-fold cross-validation. To compare the accuracy of our model, p*K*_a_ values for the whole training dataset are also predicted with PROPKA 3.1.^12^ The results obtained with the ML model, PROPKA, and the null model for GLU, ASP, and HIS are depicted in Fig. 4 (see ESI Fig. S.4† for LYS and TYR). It was found that the coefficients of determination (*r*^2^) for all amino acid types are above 0.6 with the ML model (except for LYS, *r*^2^ = 0.31) while mean absolute errors (MAEs) for all amino acid types are below 0.5 p*K*_a_ units. In the case of PROPKA, predictions have *r*^2^ < 0.3 and MAE > 0.6 with GLU and ASP being the most reliable predictions. Interestingly, PROPKA yields similar or less reliable results relative to the null model (
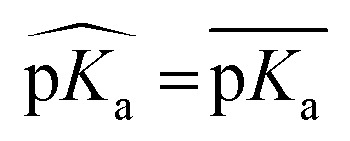
), especially for HIS, LYS and TYR. These results might be due to the PROPKA computation scheme which considers the shift of the p*K*_a_ value for the amino acid from water to protein (Δp*K*^water→protein^_a_),^11^ while the ML model is trained directly for p*K*_a_ values in the protein environment using a relatively larger training set. The number of 
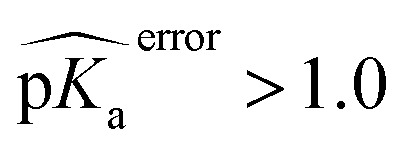
 is computed for all amino acid types (*N*_error > 1.0_) for experimental p*K*_a_ (p*K*^exp^_a_) values that are 1.0 unit below/above the p*K*_a_ value of the corresponding amino acid in water (p*K*^water^_a_). The results are depicted in Table 1. We see that the *N*_error > 1.0_ with the ML model is about twice smaller than with PROPKA for all amino acid types. These results indicate that ML model predictions are more reliable for all amino acid types that have a water to protein p*K*_a_ shift which is at least 1.0 unit (|Δp*K*^water→protein^_a_|≥ 1.0).

**Table 1:** Number of experimental p*K*_a_ values that are 1.0 p*K*_a_ unit lower or higher than the p*K*_a_ in water (*N*^exp^) and the number of prediction errors that are above 1.0 p*K*_a_ unit (*N*_error > 1.0_)

Amino acid	p*K*_a_ range	*N* ^exp^	*N*_error > 1.0_^ML Model^	*N*_error > 1.0_^Propka^
GLU	p*K*_a_ < 3.5 & p*K*_a_ > 5.5	68	12	21
ASP	p*K*_a_ < 2.8 & p*K*_a_ > 4.8	93	27	35
HIS	p*K*_a_ < 5.5 & p*K*_a_ > 7.5	85	20	55
LYS	p*K*_a_ < 9.5 & p*K*_a_ > 11.5	16	7	8
TYR	p*K*_a_ < 9.0 & p*K*_a_ > 11.0	28	0	8

**Fig. 5:**
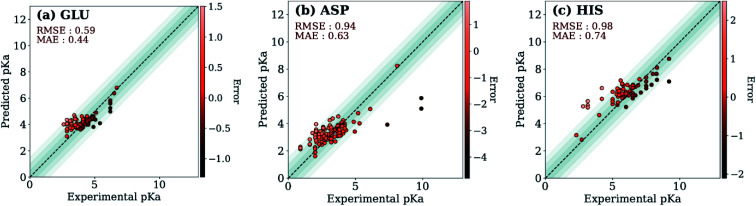
Test set predictions with ML models trained with descriptors obtained with ANI-2x.

The ML models were also evaluated with the external test dataset of p*K*_a_ values from 33 different proteins that do not appear in the training data. Results for GLU, ASP and HIS amino acids are depicted in Fig. 5 (LYS and TYR test results can be found in ESI Fig. S.5†). We found that ML models for all amino acid types provide predictions with MAE < 1.0, where GLU and LYS yield better predictions (MAE < 0.5) relative to the other amino acids. The higher MAE values, especially in the case of ASP are related to outliers that have very high/low experimental p*K*_a_ values for the corresponding amino acid (high |Δp*K*^water→protein^_a_|).

A similar evaluation was performed with DelPhiPKa^147^ using the external test set. Only 23 proteins were completed due to the extended run time over one week. Calculations are performed using default runtime parameters that are provided by the DelPhiPKa program. The RMSE/MAE values for predictions of 281 p*K*_a_ values with DelPhiPKa (present work) are computed as 1.03(0.76)/0.74(0.56), 1.17(0.60)/0.90(0.45), 1.38 (0.88)/0.96(0.67), 1.33 (0.49)/1.06(0.40), and 0.98 (0.87)/0.82(0.76) for ASP, GLU, HIS, LYS and TYR respectively. It should be noted that all calculations are performed sequentially on a linux computer with the runtime of ∼127 s/residue for DelPhiPKa and ∼0.2 s per residue for the ML model presented in this work. These results indicate that the ML model not only provides more reliable results but also runs about 500 times faster.

**Fig. 6:**
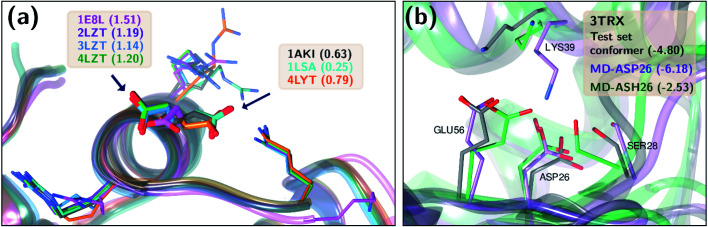
Three-dimensional representations of (a) hen lysozyme conformers in the test set with their PDB IDs. (b) thioredoxin (PDB ID: 3TRX) conformer in the test set (gray), most populated conformer obtained after molecular dynamics simulations with protonated ASP26 (purple), and the most populated conformer obtained after molecular dynamics simulations with ASP26 (green). Prediction errors for all cases are depicted within parentheses.

Two test set cases are selected to investigate the underlying reason for the errors in certain predictions: GLU7 predictions for hen egg white lysozyme conformers and ASP26 predictions for recombinant human thioredoxin conformer (Fig. 6). The hen egg lysozyme white (HEWL) test set comprises seven different crystallographic structures with multiple conformer configurations for the GLU7 residue (Fig. 6a). In all HEWL conformers, there is at least one positively charged residue within 5 Å of GLU7; ARG5 in all conformers, LYS1 in every conformer except 1 E8L, and Arg14 for all conformers except 1 E8L, 1LSA, and 4LYT. It is observed that GLU7 in three conformers (1AKI, 1LSA, and 4LYT) is in close proximity to LYS1, promoting a H-bond interaction (*R*^side chain^_GLU7–LYS1_ < 3.0 Å). In the other four HEWL conformers, there is no H-bond interaction between these residues since GLU7 is rotated to the opposite direction of the LYS1 residue. Interestingly, the prediction errors for the conformers with GLU7–LYS1 side chain interaction are lower than 1.0 while the prediction errors for the conformers that do not contain this interaction are higher than 1.0 p*K*_a_ unit. The prediction errors for the same residue with CPHMD simulations were reported to be approximately 0.8 and 1.3 with the explicit solvent and implicit solvent respectively.^45^ These results indicate that the model is highly sensitive to the conformational states of the residues and provides similar results with CPHMD simulations.

Another test case is the ASP26 on recombinant human thioredoxin (PDB IDs: 3TRX and 4TRX). Here we see prediction errors of more than 4.0 p*K*_a_ units for both conformers. The p*K*_a_ of this residue is reported as 9.9, which indicates that this residue is in the neutral form. Thus, the effect of different ASP26 states (charged and neutral) on thioredoxin is investigated with conformers obtained from molecular dynamics (MD) simulations. Since there is no distinctive conformational difference between two thioredoxin crystallographic structures, simulations were performed only with 3TRX. After 50 ns long MD simulations, the trajectories are clustered to find the most populated cluster and its representative (Fig. 6b). These representatives (negatively charged ASP: MD-ASP26, neutral ASP: MD-ASH26) are then used to predict the p*K*_a_ values of ASP26. In the case of the neutral ASP residue in the MD-ASH26 conformer, the proton on the side chain is removed before the p*K*_a_ prediction since the model is trained with negatively charged ASP. It is observed that the ASP26 conformation does not alter drastically, but the conformations of three surrounding residues (SER28, LYS39, GLU56) are affected with different ionization states of ASP. In both test set and MD-ASP26 conformers, LYS39 and GLU56 share a hydrogen bond, while this interaction does not exist in the MD-ASH26 conformer.

Additionally, the hydrogen bond interactions between ASP26 and SER28 in both test set and MD-ASP26 conformers are not observed in MD-ASH26. Instead, SER28 in MD-ASH26 forms a hydrogen bond interaction with GLU56. Predictions with the ML model reveal that the error increases with the MD-ASP26 conformer (error = 6.18) and reduces more than 1.5 units with the MD-ASH26 conformer (error = 2.53) relative to the test set conformer. These results point out the conformer sensitivity of the ML model and possible discrepancies between the crystallographic and the experimental conformers that cause the prediction error.

**Fig. 7:**
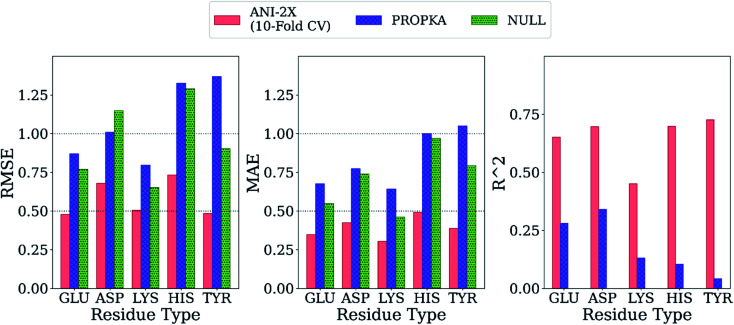
Comparison of the final model with PROPKA and null models.

Final ML models are trained using both the training and the test datasets following the same procedure for feature elimination and tests with 10-fold cross-validation. The accuracy of the predictions is compared with PROPKA and the null model. All results are depicted in Fig. 7. The RMSE values for all amino acid types are computed below 1.0 with ML models, while PROPKA predictions, except for ASP, yield higher RMSE values than the null models. Our model is found more accurate for GLU, ASP, HIS, and LYS residues relative to DelPhiPKa benchmarks without salt concentration. When the salt concentration is included in DelPhiPKa benchmarks, accuracies for LYS and ASP are comparable. Both benchmarks use a different dataset consisting 752 residues on 82 proteins.^147^ A similar pattern is observed for MAE values. Final ML models predict experimental p*K*_a_ values with MAEs below 0.5, while MAEs obtained with PROPKA predictions are substantially higher.

Interestingly, PROPKA have MAEs similar to or even worse than the null models. To our knowledge, the model presented in this work is the first empirical model that performs statistically significantly better than the null model for all titratable residues. Finally, the coefficient of determination for p*K*_a_ predictions with ML models is at least twice as large as that of PROPKA for all amino acid types.

**Fig. 8:**
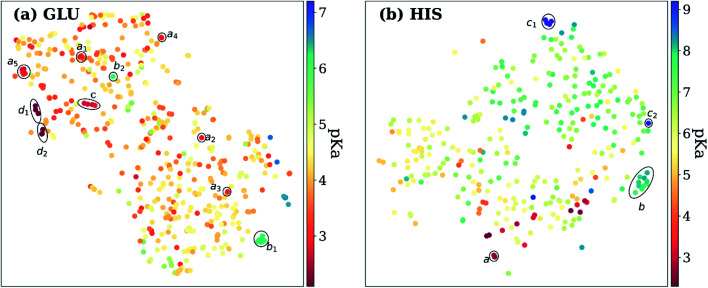
*t*-Distributed stochastic neighbor embedding (*t*-SNE) maps depicting the similarity of descriptors after recursive feature elimination for (a) GLU residues, (b) HIS residues. Each data point is colored using the color code corresponding to the experimental p*K*_a_ values.

Exploring the high dimensional p*K*_a_ training and test data in terms of similarity is impossible without dimensionality reduction. Thus, *t*-SNE^146^ is used to reduce the high dimensional data by transforming it into two-dimensional similarity maps. Such visualization allowed us to align similar residues and cross-reference them with the corresponding p*K*_a_ values. 2D *t*-SNE maps for GLU and HIS amino acids are given in Fig. 8 (see Fig. S.6† for LYS and TYR amino acids). Generally, residues with high or low experimental p*K*_a_ values are separated except for some outliers, and residues on the same class of proteins form small clusters together. For instance, GLU7 from hen egg-white lysozyme (HEWL) and turkey egg-white lysozyme (TEWL) form clusters a_i_ (Fig. 8a). Among these clusters a_5_ involves entries from both species (TEWL PDB IDs: 1LZ3, 135L and HEWL PDB IDs: 1LSA, 1LSE, 1LYS). Clusters are shown with b_i_ on Fig. 8a correspond to the GLU35 residues on HEWL and TEWL proteins. Other examples of such clusters correspond to GLU2 residues on bovine Ribonuclease A (cluster c, Fig. 8a), and GLU73 residue on Barnase (clusters d_i_, Fig. 8a). A similar pattern is observed with HIS amino acid (Fig. 8b). Residues in the same class of proteins form small clusters such as cluster a for GLU162 on Bacillus agaradhaerens family 11 xylanase, cluster b for HIS36 on myoglobin from sperm whale and horse, and clusters c_i_ for HIS72 on bovine tyrosine phosphatase.

**Fig. 9:**
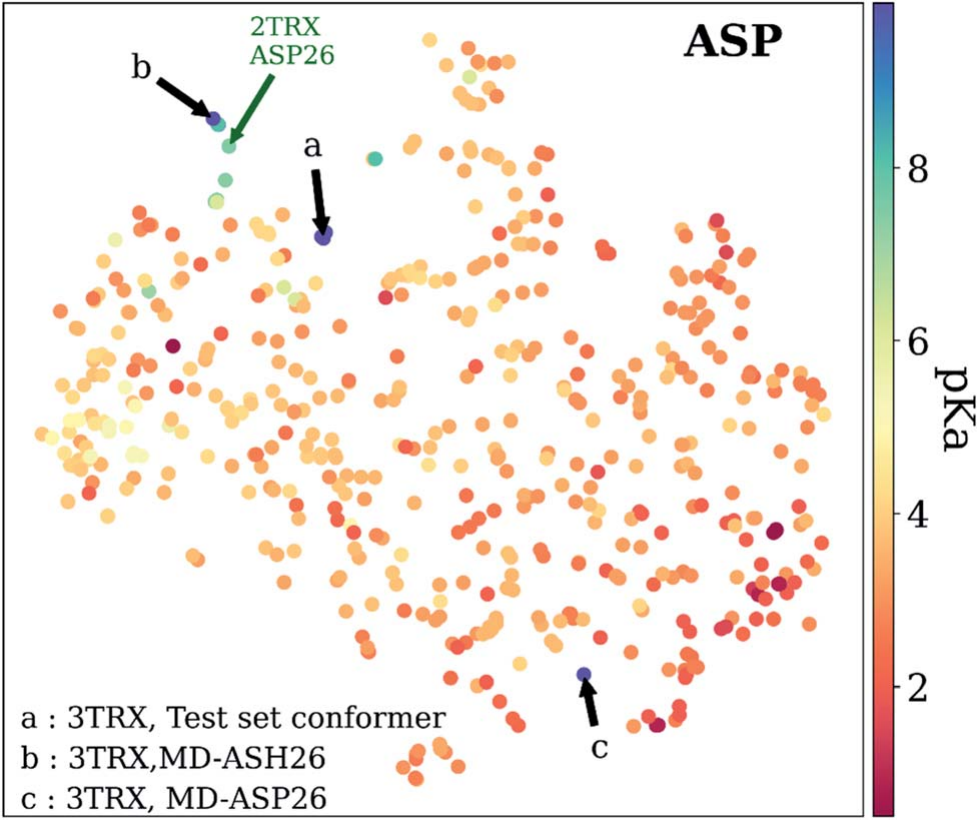
*t*-Distributed stochastic neighbor embedding (*t*-SNE) maps depicting the similarity of descriptors after recursive feature elimination for ASP residues. Conformers for 3TRX (test set conformer, conformer obtained from MD simulations with negatively charged ASP26, and neutral ASH26 side chains) are shown with arrows. Each data point is colored using the color code corresponding to the experimental p*K*_a_ values.

As mentioned before, p*K*_a_ models are sensitive to conformers, and *t*-SNE maps show some outliers. An example of such cases can be seen in Fig. 9, which depicts the *t*-SNE map for ASP amino acids. For instance, ASP26 in recombinant human thioredoxin conformer in the test set (PDB ID: 3TRX) is an outlier (arrow a on Fig. 9) on the *t*-SNE map. This point is in proximity to ASP67 on the tenth type III cell adhesion module of human fibronectin (PDB ID: 1FNA, p*K*_a_ = 4.2), ASP77 on fungal elicitor (PDB ID: 1BEG, p*K*_a_ = 2.61), and ASP28 on black rat cell adhesion molecule CD2 (PDB ID: 1HNG, p*K*_a_ = 3.57). The experimental p*K*_a_ of ASP26 on human thioredoxin is 9.9 while its neighbors have p*K*_a_ values all below p*K*_a_ = 5.0, which results in a high prediction error. The positions of residues from MD simulations (MD-ASH26: neutral ASP and MD-ASP26: negatively charged ASP) are shown with arrows b and c on Fig. 9. The *t*-SNE map shows that the MD-ASH26 conformer (arrow b) is neighboring with thioredoxin from *E.coli* (PDB ID: 2TRX, p*K*_a_ = 7.5). In contrast, the MD-ASP26 conformer (arrow c) is a neighbor to bovine ribonuclease A ASP14 (PDB ID: 3RN3, p*K*_a_ = 2.0). The error of p*K*_a_ prediction increases with the MD-ASP26 conformer and decreases with the MD-ASH26 conformer. These observations point out that the descriptors obtained from ANI-2x NNP can effectively predict the p*K*_a_ of an amino acid by describing its environment. The prediction errors are closely related to the differences in the crystal and the experimental conformers.

## Conclusion

The presented work demonstrates the capabilities of neural network potentials to provide p*K*_a_ descriptors for knowledge-based methods. The learned representation can be used to describe the chemical environment of amino acids in proteins. As the neural network potentials emerge as an alternative to the all-atom potentials, reliable p*K*_a_ descriptors can be obtained faster with their employment. The ML model presented in this work is the first empirical model that performs significantly better than the null model for all titratable residues with a runtime of ∼0.2 s per residue. The code and models are available at https://github.com/isayevlab/pKa-ANI.

A new empirical scheme for p*K*_a_ prediction of amino acids in proteins uses an ML model with descriptors calculated on ANI-2x NNP. The quantum mechanical information, which depends on the local chemical environment, is obtained from the top layers of neural network embeddings. These descriptors are used for training with the RF model to predict p*K*_a_ values. It is found that the adoption of RFE slightly improves the accuracy and yields the number of features ranging from 25 to 100 in the final model.

The accuracy of the p*K*_a_ estimations is accessed *via* 10-fold CV, and the results are compared with the null models and PROPKA predictions. It is found that the model presented in this work performs better than the null model and PROPKA. The RMSE of the p*K*_a_ predictions is below 0.7 except for HIS (0.72) with both the initial and the final models. The MAEs for all amino acid types are found below 0.5, again for the initial and the final models. In the case of PROPKA, the calculated RMSEs are over 1.0 except for GLU and LYS residues which are still over 0.7. The computed MAEs for PROPKA predictions (all above 0.6) show that PROPKA performs almost on par – if not worse – with the null model.

Further evaluations with an external test set not included in training data show a slight increase in RMSEs and MAEs. Among the external test set, two cases are selected to explore the principal reason for errors. The conformational differences of GLU7 on HEWL structures and their respective prediction errors indicate that the ML model is sensitive to the conformational differences. The latter case involves representative structures for ASP26 on recombinant human thioredoxin that are obtained with MD simulations (both with neutral and ionized ASP26 side chain). The p*K*_a_ predictions with these representatives confirm the conformational sensitivity of the ML model. Conceptually, a protein p*K*_a_ predictor should be sensitive to conformational alterations. Two test cases demonstrate the capability of the ML model in distinguishing different conformational states. Therefore, the errors obtained with the presented models are closely related to the conformational discrepancies between the crystal (fixed) and experimental (flexible) structures.

As with any model, the present approach has limitations. Some of them, such as the absence of Cys and Ser, can be overcome by adding more training data, and mining p*K*_a_ values from the primary literature. Future work will aim to extend the present model for coenzyme and cofactor effects. The current ANI descriptor has only biogenic elements and has not parametrized for metals, therefore all HETATM entries in PDB files are ignored. There is a set of limitations that would require the development of a new approach, for instance inclusion of the ionic strength or different solvents into NN descriptors.

## Data availability

The code and ML models are available at https://github.com/isayevlab/pKa-ANI.

## Author contributions

O. I. conceived the idea. H. G. carried out method implementation and performed all calculations. All authors critically contributed to the design of the project, analysis of results, and writing of the manuscript. O. I. supervised and acquired funding for the project.

## Conflicts of interest

There are no conflicts to declare.

## Supplementary Material

SC-013-D1SC05610G-s001
