# Supplementary material for: Prediction of protein pKa with representation learning
Source: Chem Sci. 2022 Feb 1;13(8):2462–74. doi: 10.1039/d1sc05610g (PMC8864681; doi:10.1039/d1sc05610g)
Supplement: SC-013-D1SC05610G-s001 [file SC-013-D1SC05610G-s001.pdf]

Electronic Supplementary Material (ESI) for Chemical Science.
This journal is © The Royal Society of Chemistry 2022

Supporting Information

for

Prediction of Protein pKa with Representation Learning

Hatice Gokcan, Olexandr Isayev*

Department of Chemistry, Mellon College of Science,

Carnegie Mellon University, Pittsburgh, PA

[*] Correspondence: olexandr@olexandrisayev.com

Table of Contents

Figure S.1 Distribution of experimental pKa values for (a) GLU, (b) ASP, (c) LYS, (d) HIS, and (e) TYR amino acids within the dataset. Individual number of entries in the training and external test sets are depicted within parentheses as (training set size, external test set size). 3

Figure S.2 Flowchart for data curation and descriptor calculations prior to training. 4

Figure S.3 Comparison of different machine learning algorithms and the effect of recursive feature elimination. 4

Figure S.4 The accuracy of the predictions of experimental pKa values for a) 10-fold cross validation predictions with ML model for LYS, b) 10-fold cross validation predictions with ML model for TYR, c) LYS using Propka, d) TYR using Propka, e) LYS with Null model, f) TYR with Null model. 5

Figure S.5 Test set predictions with ML models trained with descriptors obtained with ANI-2x. ..6

Figure S.6 t-Distributed stochastic neighbor embedding (t-SNE) maps depicting similarity of descriptors after recursive feature elimination for (a) LYS residues, (b) TYR residues. Each data point is colored by the corresponding experimental pKa values 6

Figure S.7 The accuracy of the predictions of experimental pKa values with final model for a) 10-fold cross validation predictions with ML model for GLU, b) 10-fold cross validation predictions with ML model for ASP, c) 10-fold cross validation predictions with ML model for HIS, d) GLU using Propka, e) ASP using Propka, f) HIS using Propka, g) GLU with Null model, h) ASP with Null model, i) HIS with Null model. 7

Figure S.8 The accuracy of the predictions of experimental pKa values with final model for a) 10-fold cross validation predictions with ML model for LYS, b) 10-fold cross validation predictions with ML model for TYR, c) LYS using Propka, d) TYR using Propka, e) LYS with Null model, f) TYR with Null model. 8

Figure S.9 Descriptors for pKa predictions of Asp amino acid and their importance that are obtained after RFE procedure. 9

Figure S.10 Descriptors for pKa predictions of Glu amino acid and their importance that are obtained after RFE procedure. 10

Figure S.11 Descriptors for pKa predictions of His amino acid and their importance that are obtained after RFE procedure. 11

Figure S.12 Descriptors for pKa predictions of Lys amino acid and their importance that are obtained after RFE procedure. 12

Figure S.13 Descriptors for pKa predictions of Tyr amino acid and their importance that are obtained after RFE procedure. 13

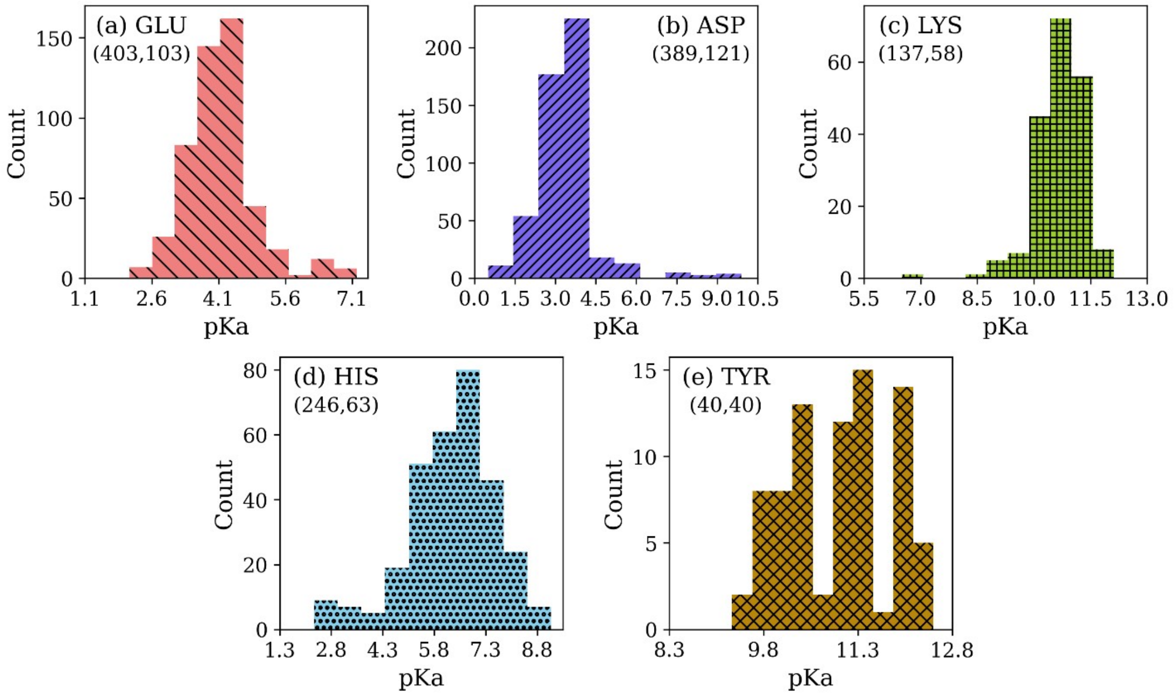

Figure S.1 Distribution of experimental pKa values for (a) GLU, (b) ASP, (c) LYS, (d) HIS, and (e) TYR amino acids within the dataset. Individual number of entries in the training and external test sets are depicted within parentheses as (training set size, external test set size).

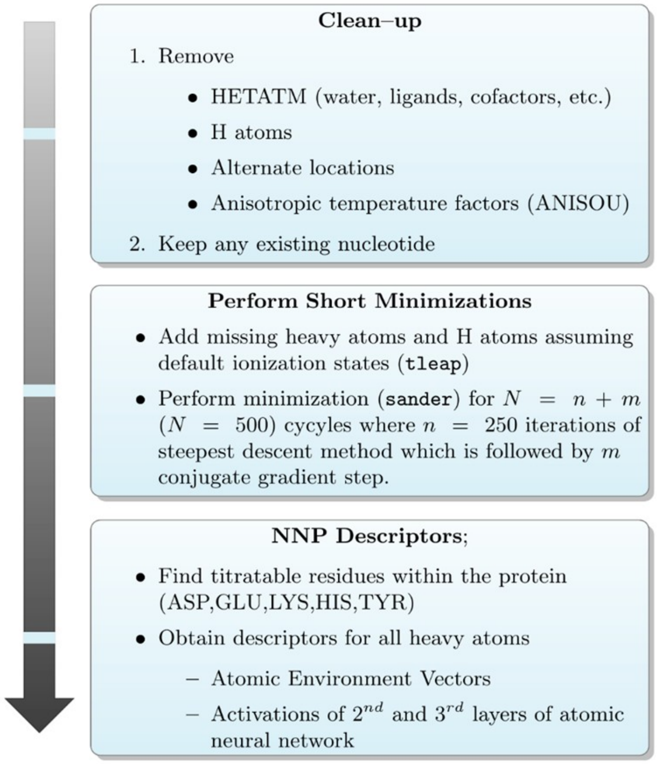

Figure S.2 Flowchart for data curation and descriptor calculations prior to training.

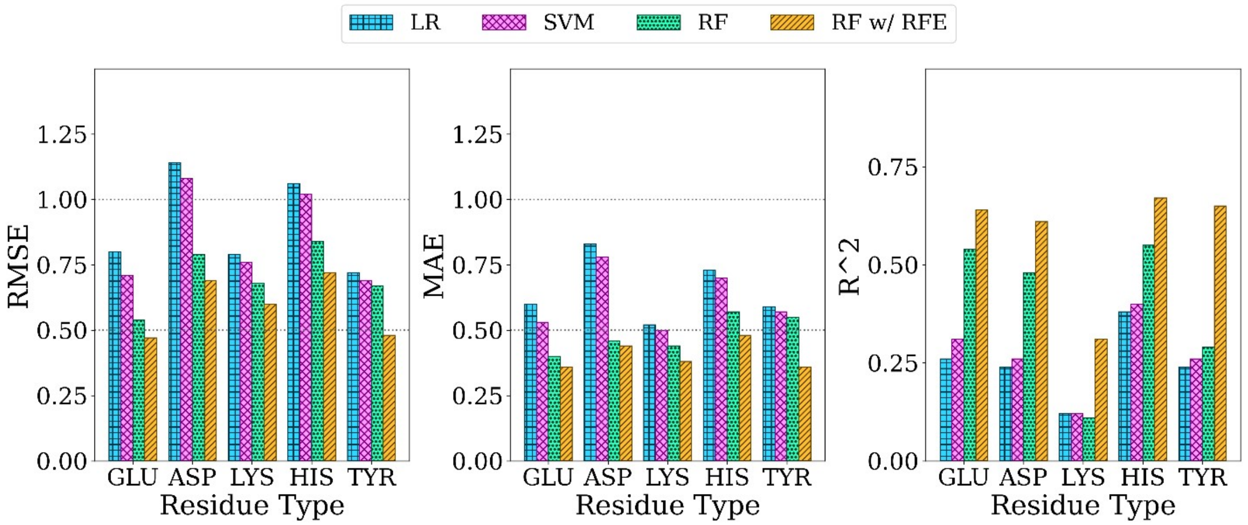

Figure S.3 Comparison of different machine learning algorithms and the effect of recursive feature elimination.

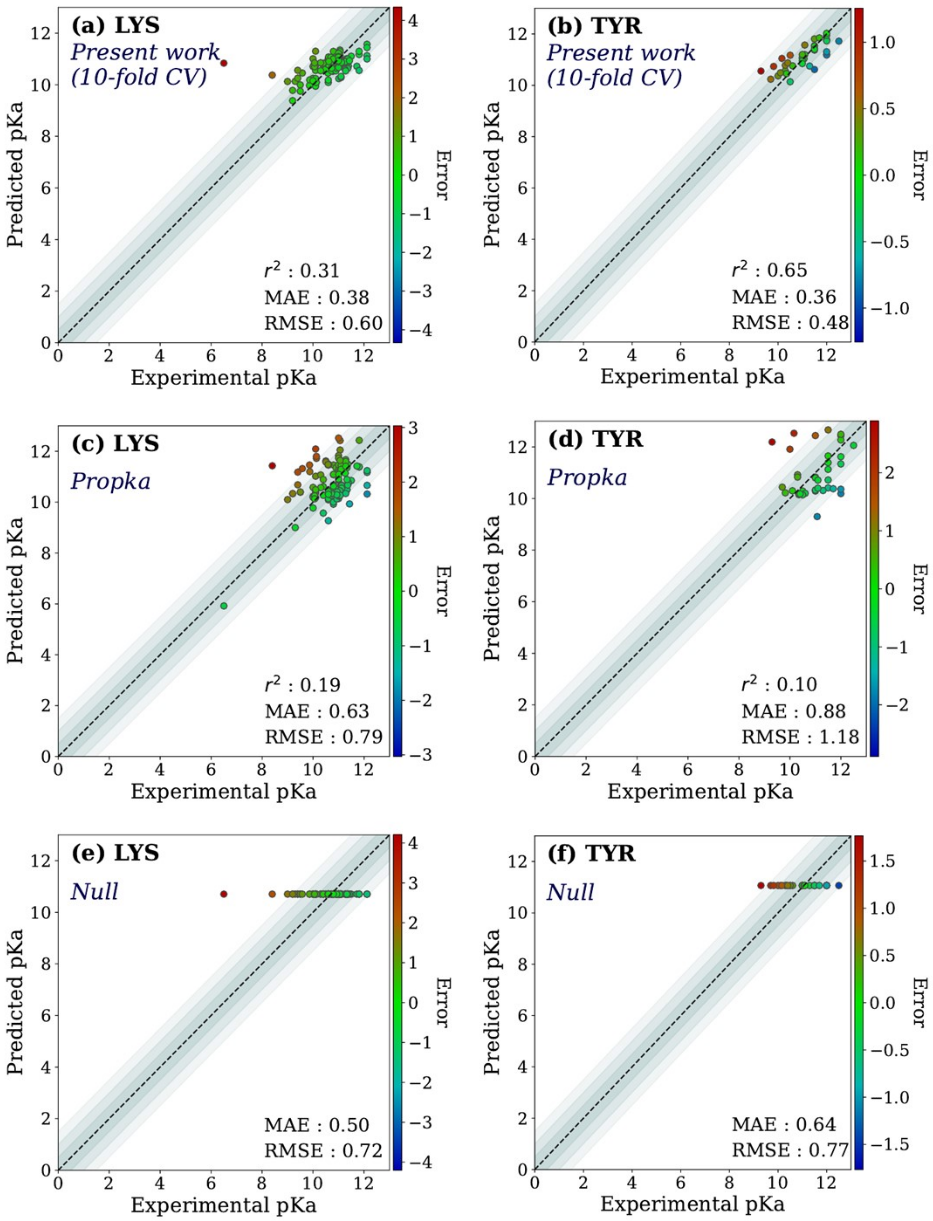

Figure S.4 The accuracy of the predictions of experimental pKa values for a) 10-fold cross validation predictions with ML model for LYS, b) 10-fold cross validation predictions with ML model for TYR, c) LYS using Propka, d) TYR using Propka, e) LYS with Null model, f) TYR with Null model.

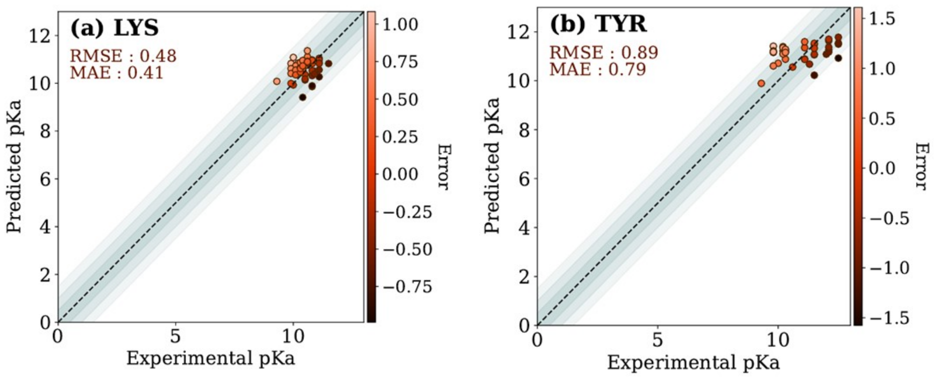

Figure S.5 Test set predictions with ML models trained with descriptors obtained with ANI-2x.

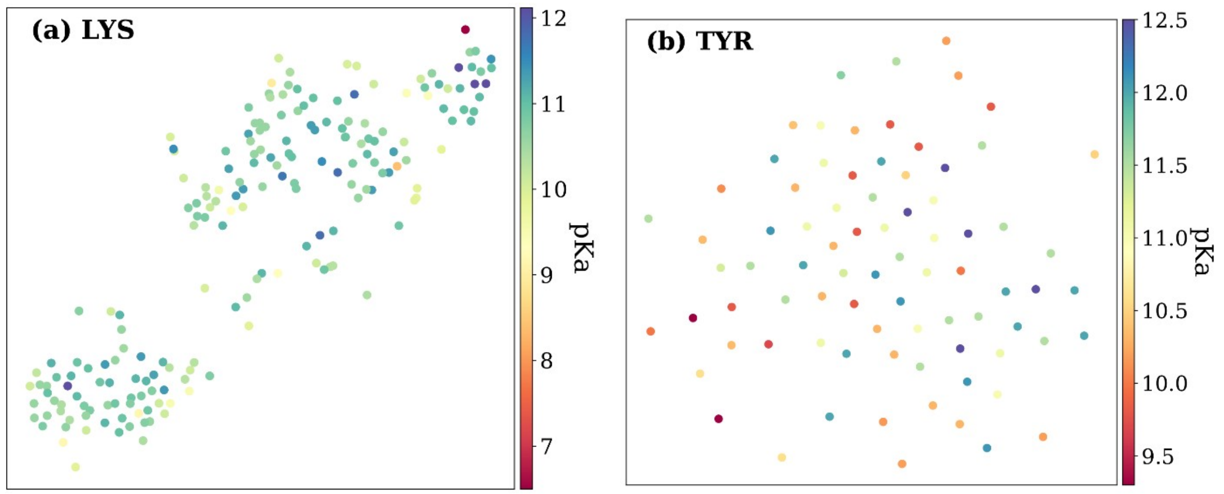

Figure S.6 t-Distributed stochastic neighbor embedding (t-SNE) maps depicting similarity of descriptors after recursive feature elimination for (a) LYS residues, (b) TYR residues. Each data point is colored by the corresponding experimental pKa values

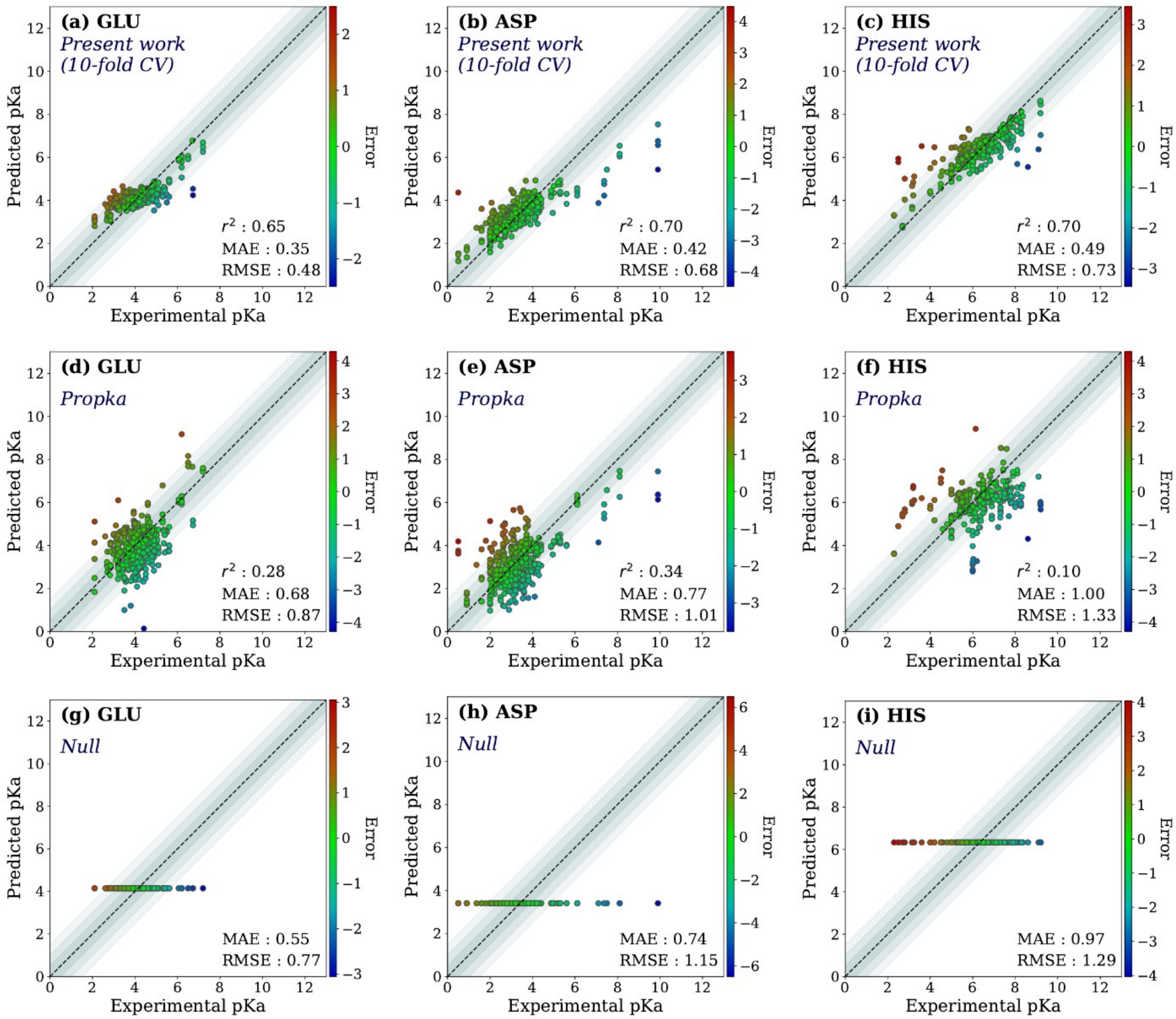

Figure S.7 The accuracy of the predictions of experimental pKa values with final model for a) 10-fold cross validation predictions with ML model for GLU, b) 10-fold cross validation predictions with ML model for ASP, c) 10-fold cross validation predictions with ML model for HIS, d) GLU using Propka, e) ASP using Propka, f) HIS using Propka, g) GLU with Null model, h) ASP with Null model, i) HIS with Null model.

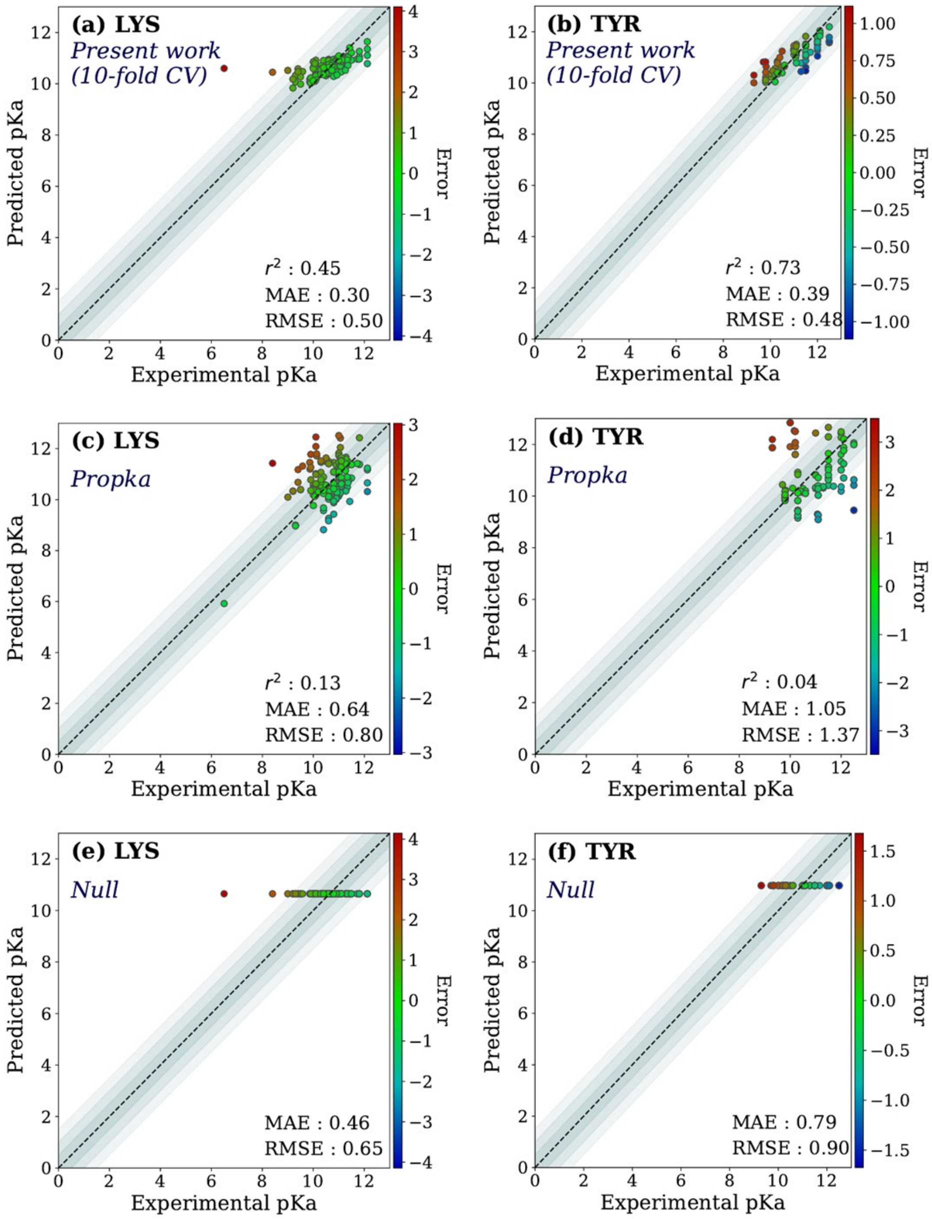

Figure S.8 The accuracy of the predictions of experimental pKa values with final model for a) 10-fold cross validation predictions with ML model for LYS, b) 10-fold cross validation predictions with ML model for TYR, c) LYS using Propka, d) TYR using Propka, e) LYS with Null model, f) TYR with Null model.

.

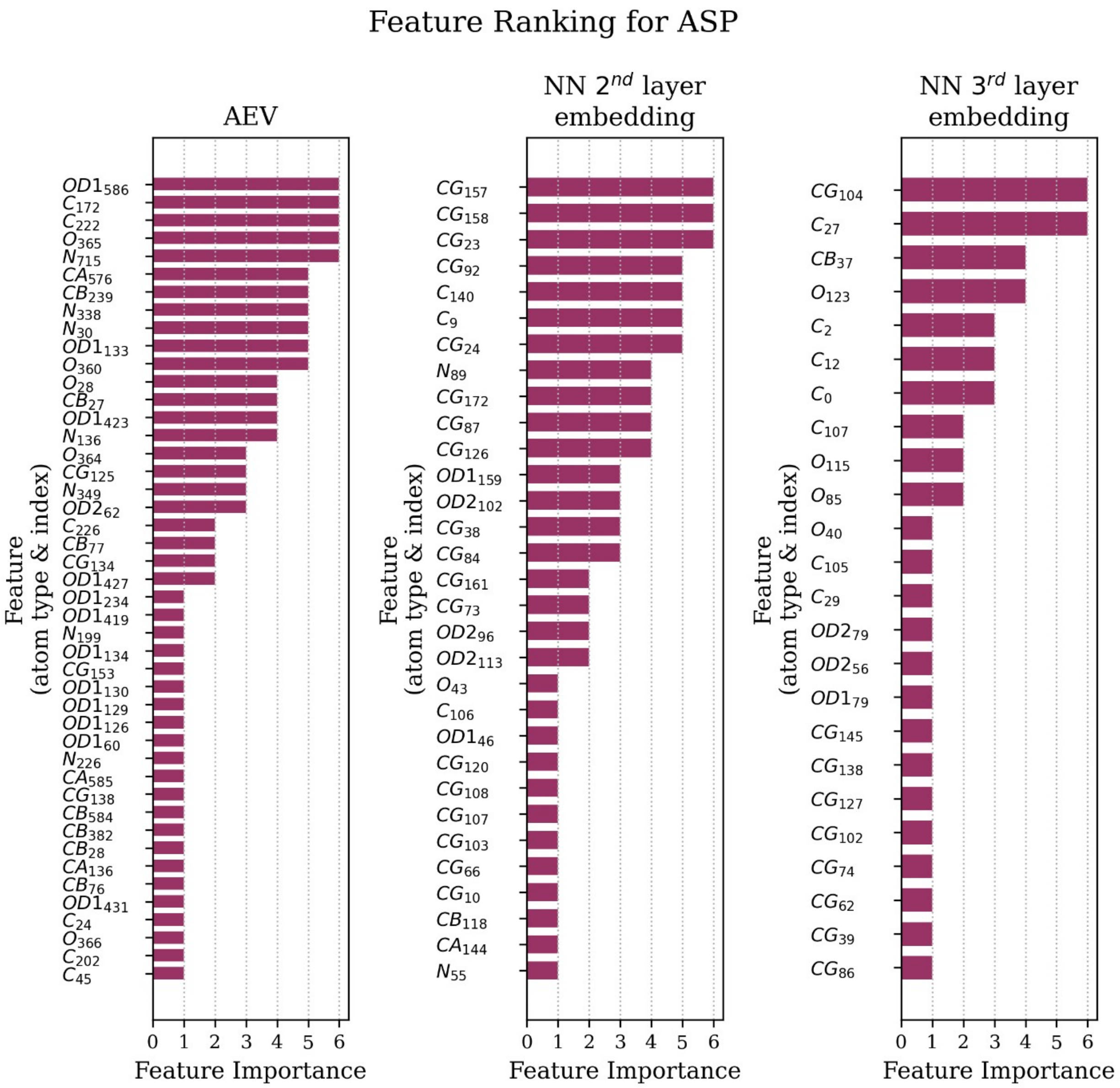

Figure S.9 Descriptors for pKa predictions of Asp amino acid and their importance that are obtained after RFE procedure.

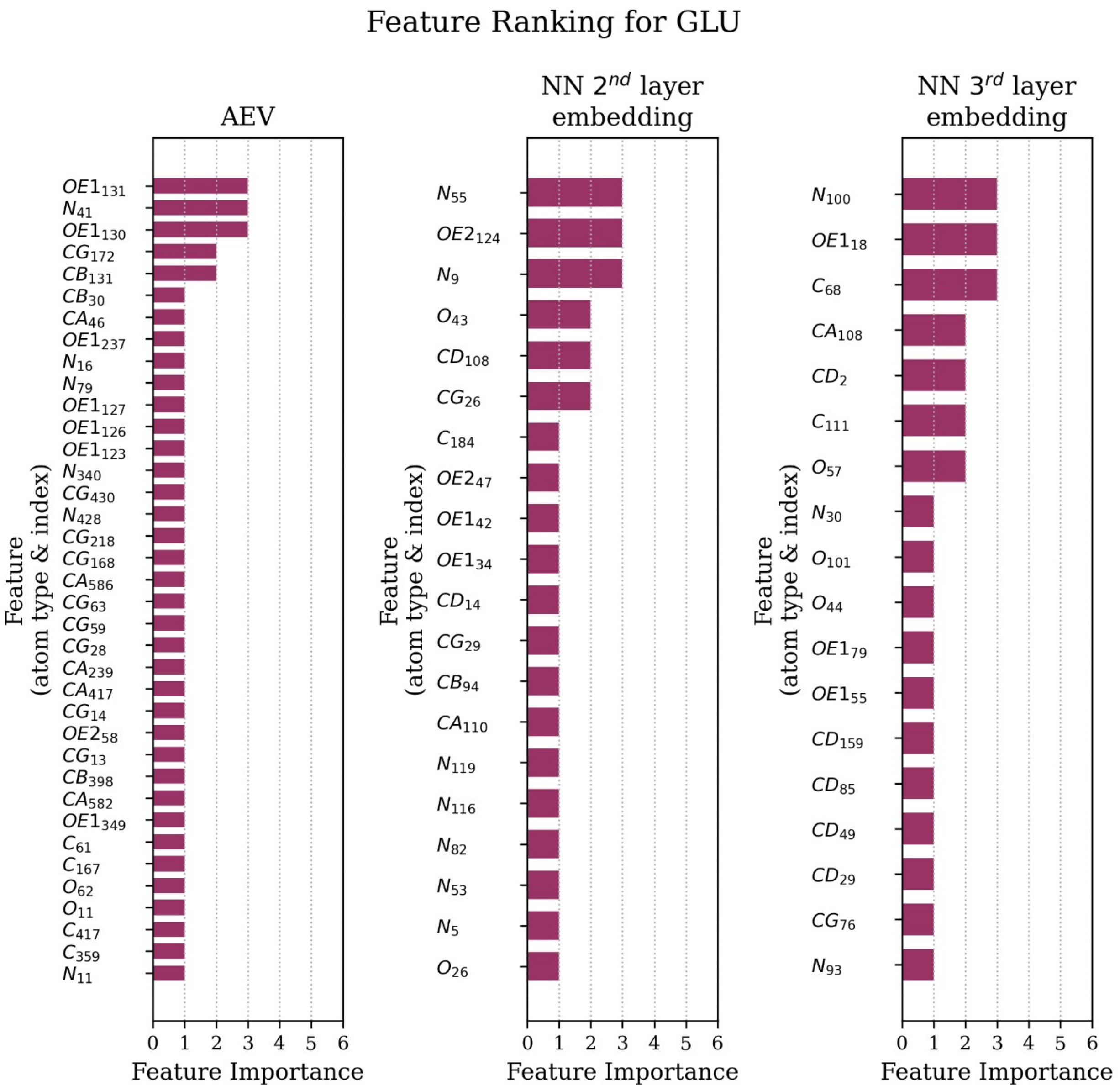

Figure S.10 Descriptors for pKa predictions of Glu amino acid and their importance that are obtained after RFE procedure.

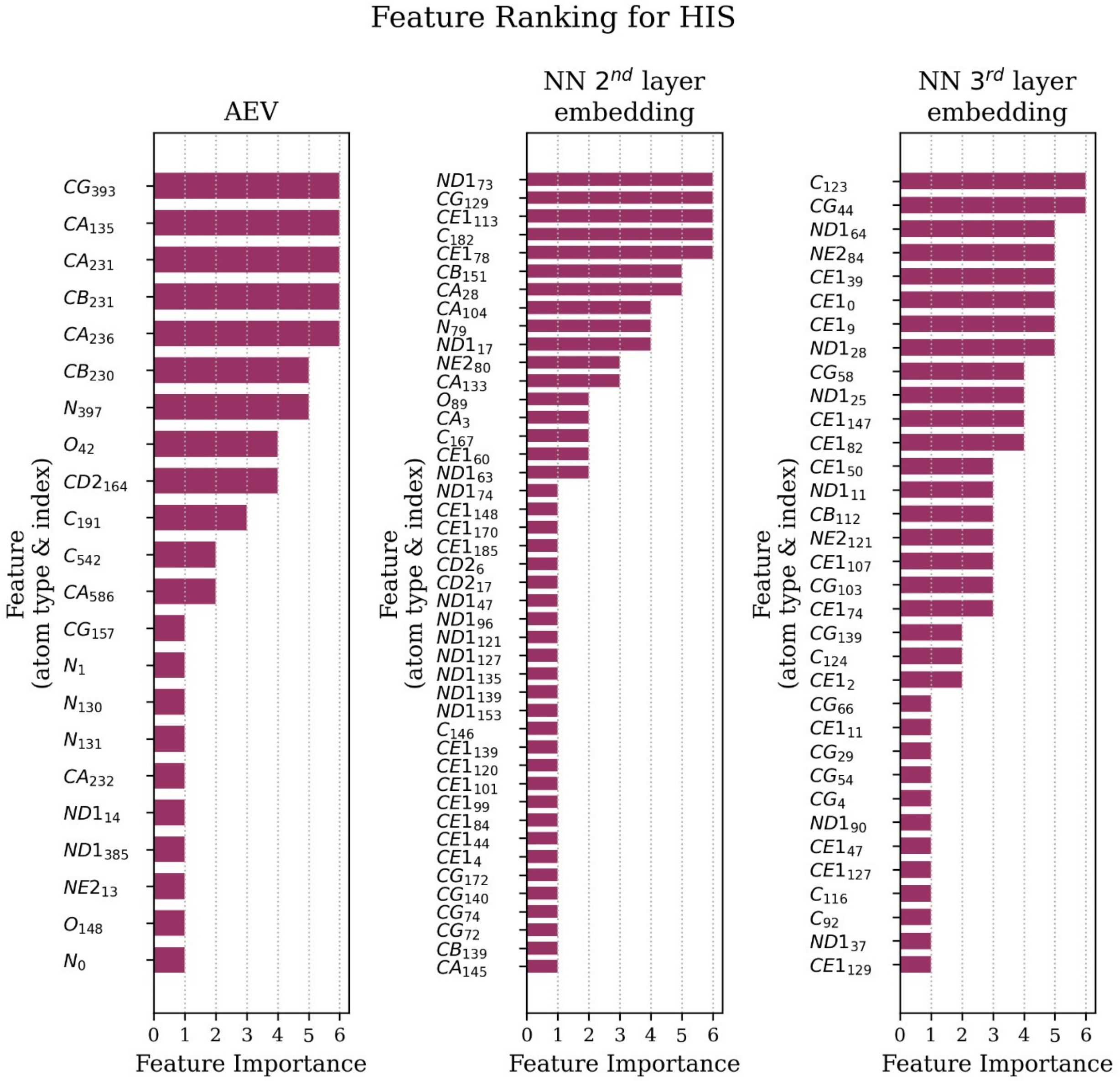

Figure S.11 Descriptors for pKa predictions of His amino acid and their importance that are obtained after RFE procedure.

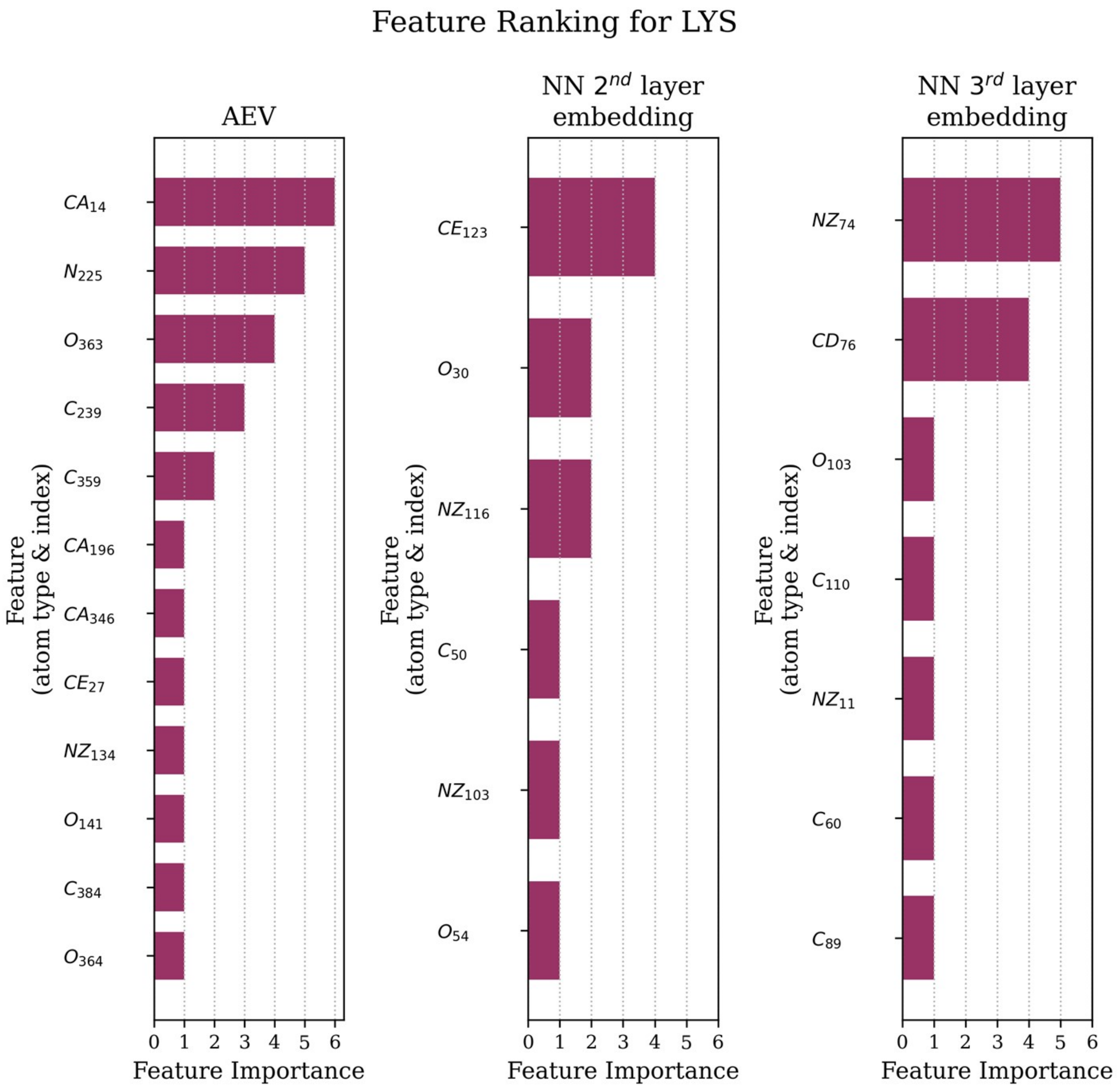

Figure S.12 Descriptors for pKa predictions of Lys amino acid and their importance that are obtained after RFE procedure.

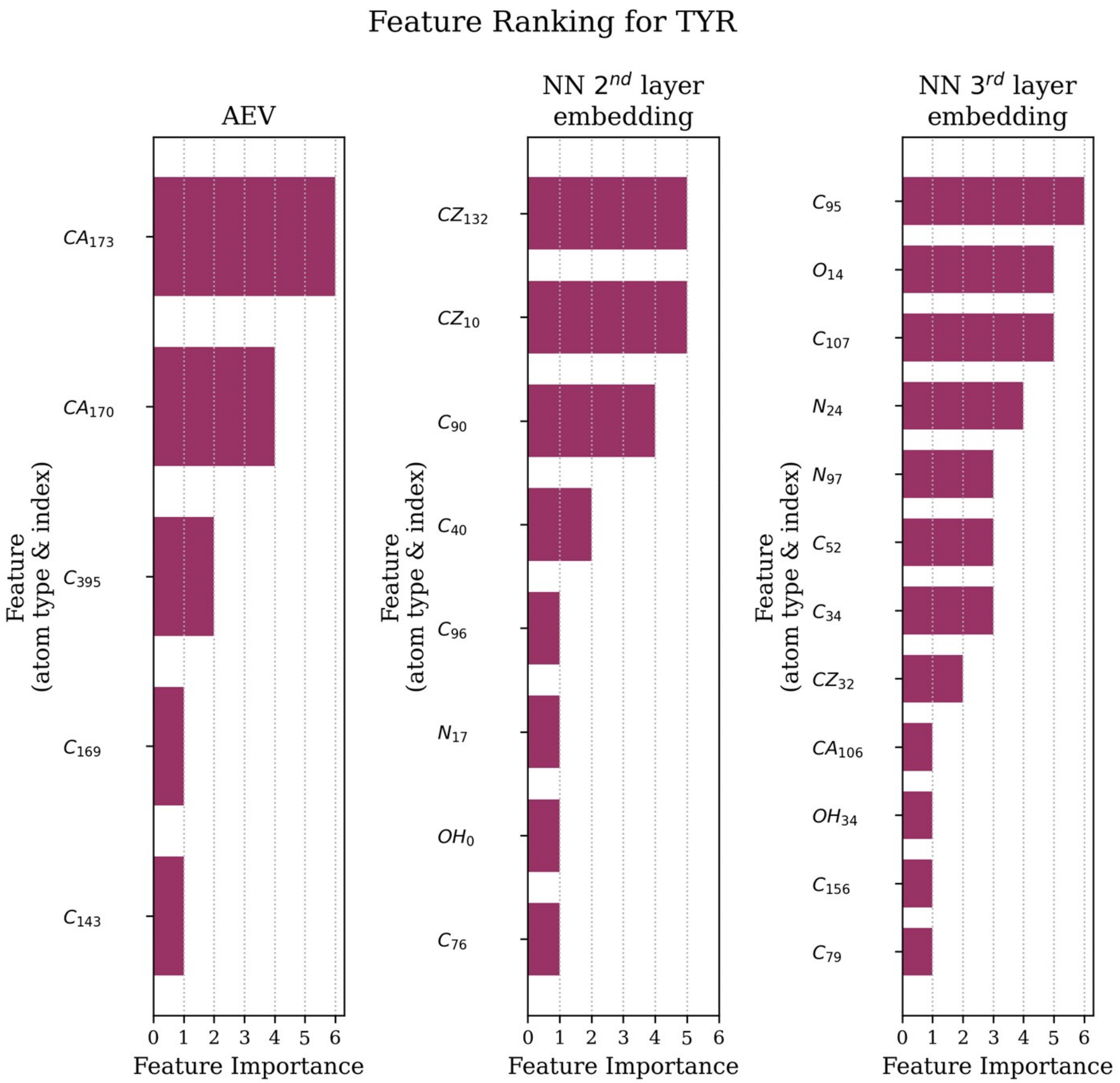

Figure S.13 Descriptors for pKa predictions of Tyr amino acid and their importance that are obtained after RFE procedure.
